# Implementation of Synoptic Reporting for Endoscopic Localization of Complex Colorectal Neoplasms

**DOI:** 10.7759/cureus.54480

**Published:** 2024-02-19

**Authors:** Haven Roy, Garrett Johnson, Harminder Singh, Eric Hyun, Dana Moffatt, Ashley Vergis, Ramzi Helewa

**Affiliations:** 1 Surgery, University of Manitoba, Winnipeg, CAN; 2 Internal Medicine, University of Manitoba, Winnipeg, CAN

**Keywords:** colorectal neoplasm, synoptic reporting, documentation, tattooing, colonoscopy

## Abstract

Introduction

Lack of documented tattooing of colorectal neoplasms at index colonoscopy results in high repeat preoperative colonoscopy rates. We developed national consensus recommendations for endoscopic localization and piloted an electronic synoptic reporting template. We report on the implementation and perceptions of using synoptic reporting to enhance colorectal lesion marking in a central Canadian healthcare system.

Methods

We implemented the template within our endoscopy reporting system and ran an infographic education campaign. We then conducted a follow-up email-based interview with all regional endoscopists. Thematic analysis and a mixed-methods triangulation approach were employed to synthesize qualitative and quantitative data.

Results

The interview was completed by 28/52 endoscopists (54%). Most (60.7%; n = 17) completed >100 colonoscopies and 71.4% (n = 20) identified six to 20 neoplasms requiring tattooing since introduction. A total of 50% (n = 14) used the template. Those not using it were unaware of it (42.9%; n = 12), or preferred using narrative text (17.9%; n = 5). Users reported modest mean functionality scores (intuitiveness: 3.56/5; efficiency: 3.7/5) and high impact scores (credible: 4.22/5; informative: 4.21/5). However, the perception of the synoptic template’s ability to reduce the repeat preoperative colonoscopy rate was more circumspect (3.76/5).

Conclusions

Endoscopists believed the synoptic template was a functional, impactful tool that would improve communication and help to decrease the repeat preoperative colonoscopy rate. However, synoptic template uptake was limited by provider awareness, therefore more educational efforts are needed to increase uptake.

## Introduction

Widespread adoption of minimally invasive techniques for oncologic colorectal surgery has dramatically increased the importance of appropriate preoperative endoscopic tattoo localization and electronic documentation of complex neoplasms of the colon and rectum. Appropriate employment of preoperative endoscopic tattooing allows for intra-operative localization of the lesion of interest in greater than 95% of cases [1-4]. However, failure to appropriately localize a lesion endoscopically with the tattoo, or to appropriately document characteristics of the lesion and tattoo placement, leads to increased rates of repeat endoscopy, conversion from minimally invasive surgery (MIS) to open surgery, changes to operative conduct, increased operative time, and even incorrect segmental resection [5-7]. Despite the well-established importance of preoperative tumor marking, practice patterns with respect to tattoo placement and documentation continue to vary widely in the literature [8,9].

Our group has undertaken a large retrospective review of over 1000 patients with advanced colorectal neoplasms requiring surgical resection and demonstrated that only 57% of these patients received tattoo localization at the time of index endoscopy. In the majority of cases (67%), the presence or location of that tattoo was not documented. Insufficient tattoo localization and communication resulted in a repeat preoperative colonoscopy rate of 29% over a 10-year period, delaying surgery and exposing patients to unnecessary discomfort, risks, and costs to the healthcare system [10].

We identified two key target areas for quality improvement in this area. First, despite the importance of the subject, evidence-based recommendations regarding endoscopic localization and documentation of advanced colorectal neoplasms had been lacking within the literature until recently. A large knowledge gap exists and practice patterns vary accordingly [1-9]. In order to correct this knowledge gap, Johnson and colleagues performed a systematic review of existing endoscopy guidelines and a thorough narrative review of endoscopy-related literature to identify existing global practice recommendations. They then used the Delphi model to develop national multidisciplinary consensus-derived recommendations for optimal endoscopic localization and documentation of advanced colorectal neoplasms [11].

Following recommendation development, we performed a qualitative interview study and a needs assessment, using a mainstream implementation science framework called the Consolidated Framework for Implementation Research [12,13]. We interviewed regional endoscopists to identify barriers and facilitators to following the new recommendations. Based on this research, we identified a list of priority items, including the need to change medical record systems and promote awareness of the new recommendations with an educational campaign [12]. 

Synoptic reporting has been used extensively as a key tool for increasing accurate and effective written communication between multidisciplinary care teams [14]. We hypothesized that introducing synoptic reporting of colorectal tattoo localization would provide a technological solution to the communication gap that existed between endoscopists and surgeons, as well as an educational tool for the dissemination of the new practice recommendations. The aim of this study is to examine the implementation and use of synoptic reporting of advanced colorectal neoplasm endoscopic localization in a tertiary referral setting.

## Materials and methods

Synoptic reporting

We partnered with industry experts to design and introduce a synoptic reporting template to the Winnipeg Regional Health Authority (WRHA) electronic endoscopy reporting system (EndoSoft® LLC. EndoVault for Windows 2021. Schenectady, NY). The existing software included a predesigned synoptic template for the characterization of polyps. We updated this template to include options that reflected the new consensus recommendations for localizing advanced colorectal neoplasms [11]. Specifically, options were introduced to document whether a tattoo was placed and to indicate how many spots were injected (either in one location or three quadrants, depending on neoplasm characteristics). Options were also made available to document the orientation of the tattoo with respect to the lesion (2-3 cm distal to the polyp). If a tattoo was not placed or the orientation of the tattoo did not conform to consensus recommendations, this could also be recorded on the template. Use of the synoptic template was optional, and the reporting software also provides an option for narrative reporting. We advertised this intervention with a region-wide email distribution, as well as a concurrent infographic printout posted in every endoscopy suite in the region. The notices detailed the synoptic reporting updates, and the infographic reinforced the synoptic template by reviewing recommendations for indications, technique, and documentation for the endoscopic tattooing of advanced colorectal neoplasms. 

Study design 

To gather detailed perspectives on the functionality of the synoptic template and the influence it would have on the clinical management of advanced colorectal neoplasms, we designed an internet-based survey which we piloted among the study authors (all of whom are practicing endoscopists) and revised according to their feedback. Five months after implementing the synoptic template locally, we distributed the survey via a secure web-based platform (SurveyMonkey Inc. SurveyMonkey Premium Edition. San Mateo, CA) to every practicing endoscopist within the WRHA in Winnipeg, Manitoba, Canada. The survey included demographics, pre- and post-implementation volume data, quantitative measurements, as well as qualitative, open-ended narrative feedback opportunities. In order to avoid a central tendency of responses to Likert scale questions, we accompanied these questions with written example responses, with 1 being a strongly negative or minimal response (i.e., "they should never be used"), 3 being intermediate (i.e., "they should sometimes be used"), and 5 being a strongly positive or maximum response (i.e., "they should always be used"). 

Data analysis

We used a thematic analysis approach to organize and transform qualitative survey data into themes [15,16]. We then identified common themes related to the functionality and anticipated effect of our intervention and compared them to quantitative survey data using a concurrent triangulation approach [17]. Standard descriptive statistical analysis was performed on all quantitative data.

## Results

The interview was distributed to all 52 practicing endoscopists in the WRHA and was completed by 28 participants, for a response rate of 54% (Table 1). A total of 54% of respondents were surgeons (n = 15) and 46% were gastroenterologists (n = 13). A total of 78% of respondents were male (n = 22), 18% were female (n = 5), and 4% preferred not to state their gender (n = 1). Prior to implementing the synoptic template, the majority of respondents (57%; n = 16) never or only rarely made use of the polyp identification templates that were available within the software. Most respondents (61%; n = 16) completed greater than 100 colonoscopies after introducing the synoptic template. The majority of respondents (71.4%; n = 20 ) stated that they had identified between six and 20 advanced neoplasms requiring tattoo placement since the release of the new recommendations, while 21.4% (n = 6) had identified fewer than that (between one and five polyps) and 7.2% (n = 2) had identified more than 20. Half of the respondents (n = 14) stated that they made no use of the synoptic template, while only 32% (n = 9) stated that they had made regular use of the templates, and 18% (n = 5) said they had sometimes used them.

**Table 1:** Participant self-reported demographic characteristics and endoscopy report usage All figures show the number of total respondents with the percentage of the total in brackets, except where indicated.

Participant characteristics	All participants (n = 28)	Gastroenterologists (n = 13)	Surgeons (n = 15)
Median years in practice (range)	21+ (1-21+)	21+ (1-21+)	11-15 (1-21+)
Gender			
Male	22 (78)	12 (92)	10 (66.7)
Female	5 (18)	0 (0)	5 (33.3)
Undisclosed	1 (4)	1 (8)	0 (0)
Frequency participants used other colonoscopy documentation templates prior to the implementation of the new synoptic report			
Always	4 (14)	2 (15.4)	2 (13.3)
Often	6 (22)	3 (23)	3 (20)
Sometimes	2 (7)	2 (15.4)	0 (0)
Rarely	5 (18)	0 (0)	5 (33.3)
Never	11 (39)	6 (46.2)	5 (33.3)
Colonoscopies performed post-implementation of the new synoptic report			
1-25	0 (0)	0 (0)	0 (0)
26-50	3 (11)	1 (8)	2 (13)
51-75	4 (14)	1 (8)	3 (20)
76-100	4 (14)	1 (8)	3 (20)
>100	17 (61)	10 (76)	7 (47)
Advanced neoplasms identified post-implementation of the new synoptic report			
1-5	6 (21.4)	2 (15.4)	4 (26.7)
6-10	13 (46.4)	5 (38.4)	8 (53.3)
11-20	7 (25)	4 (30.8)	3 (20)
>20	2 (7.2)	2 (15.4)	-
Synoptic template usage post-implementation			
Always	9 (32)	3 (23)	6 (40)
Sometimes	5 (18)	3 (23)	2 (13)
Never	14 (50)	7 (54)	7 (47)

Narrative responses were requested regarding barriers to synoptic template use from November 2022 until January 2023. Thematic analysis of responses revealed that “lack of awareness” was the primary explanation for low uptake, as nearly half (42.9%; n = 12) of responses fit within this theme. A secondary theme that was identified in 18% (n = 5) of the responses was "click fatigue” in which respondents expressed a preference toward free text data entry in order to minimize selecting additional boxes within the synoptic reporting templates. For example, Participant 20 stated, “I type all of my info to try to minimize clicking boxes.” Similarly, Participant 25 explained, “I find the point and click menus limiting and do not typically use them.” Participant 18 said, “I find templates to be a waste of time and I don’t use them for any of my reporting.” Participant 4 said, “It’s faster to type it myself.”

**Figure 1:**
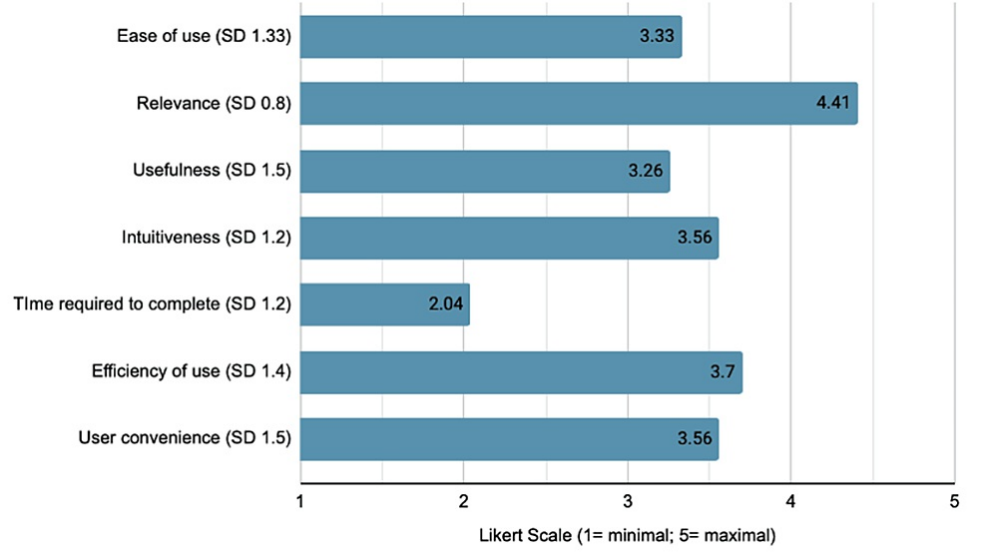
Mean Likert questionnaire scores of participant-reported functionality of the new consensus recommendations in the new synoptic reporting template SD, standard deviation

Figure 1 summarizes respondent perspectives according to Likert scale questions regarding the functionality of the new consensus recommendations in the new synoptic reporting template. Respondents reported modest ease of use (3.33/5), intuitiveness (3.56/5), and efficiency scores (3.7/5). They felt the synoptic template would take very little time to complete in the midst of a busy endoscopy schedule (2.04/5; low score reflects less time). They felt most strongly that this recommendation-based synoptic template was relevant to their field of practice (4.41/5).

**Figure 2:**
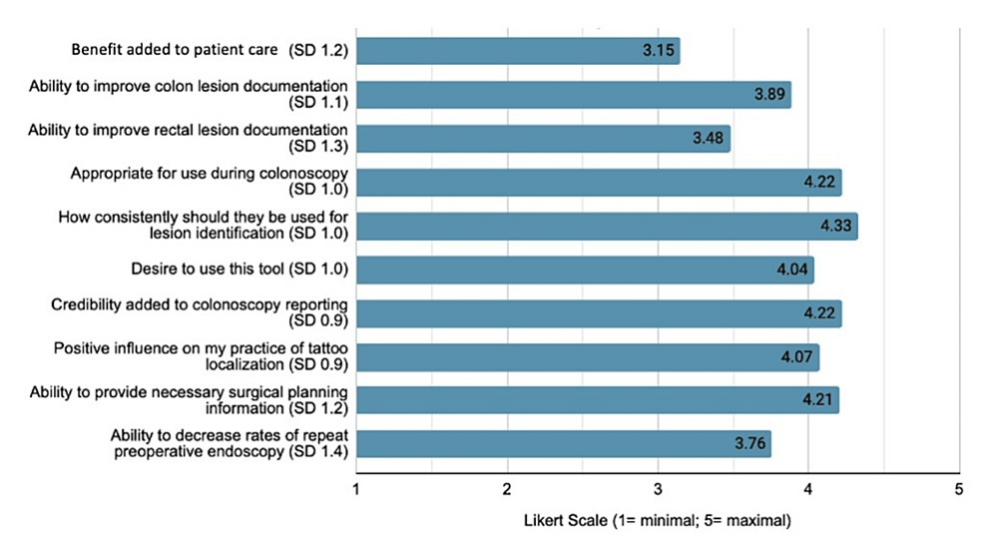
Mean Likert questionnaire scores of the participant-reported impact of the synoptic template on clinical practice and interdisciplinary communication SD, standard deviation

Figure 2 summarizes Likert scale responses on the perceived impact of the synoptic template on clinical practice and interdisciplinary communication. Very high appropriateness (4.22/5), credibility added (4.22/5), and positive influence (4.07/5) scores were reported. Respondents felt that this format would improve colon lesion documentation more so than rectal (3.89/5 and 3.48/5, respectively), and, if widely used, there was a strong assertion that synoptic reporting would provide the necessary information for efficient surgical planning (4.21/5). Interestingly, they felt less strongly about the ability of this template to ultimately decrease the rate of repeat preoperative endoscopy (3.76/5) and to add true benefit to patient care (3.15/5). Nevertheless, they still expressed a strong desire to make use of this updated tool (4.04/5).

These concepts are reinforced in the thematic analysis of narrative responses received regarding methods for improving the functionality and impact of the synoptic template. The most prominently identified theme was the need for expanded advertising to raise awareness (25% of respondents; n = 7). A total of 46.4% of respondents (n = 13) indicated that the best method for expanded advertisement would be via email, while 25% (n = 7) felt provincial endoscopy rounds would be most appropriate to accomplish this goal. Another common theme identified was the need for further technological improvement (21.4% of respondents; n = 6). Examples in this category focused on the need to integrate free text options into mandatory synoptic templating to allow expanded detail as necessary.

## Discussion

The compiled data from this study provides us with valuable insight into the benefits and limitations of synoptic templating for endoscopic tattoo localization as we devise strategies for broader applications. The response rate of 54% (28 of 52 polled endoscopists), roughly evenly split between surgeons and gastroenterologists, gave us a fair representation of a regional cohort. Particularly with respect to the qualitative data in this study, smaller sample sizes of approximately 20 are common and preferred for gaining insight that would not be possible with a larger number of subjects [15]. Most respondents had a great deal of endoscopy experience, with a median time in practice of greater than 21 years. Furthermore, the majority of the endoscopists who completed our study performed over 100 colonoscopies post-implementation and identified at least five advanced colorectal neoplasms requiring tattoo placement since the campaign began, so they had ample opportunity to interact with the new synoptic template and recommendations.

The strength of this study is its demonstration that these new consensus recommendations, and their application within a synoptic reporting template, resonate strongly with endoscopists who participated. The high rates of perceived impact (i.e., appropriateness, positive impact, and ability to improve documentation; Figure 2) reflect this sentiment. Furthermore, respondents felt the synoptic template was also functional and practical in terms of a real-life option for appropriately documenting advanced colonic lesions (Figure 1). They reported moderate ease of use, intuitiveness, and efficiency scores, and they felt the time investment required to perform this documentation would be minimal.

In prior work, the observed 29% repeat preoperative endoscopy rate was partially attributed to a lack of effective communication between endoscopist and surgeon regarding the presence and orientation of tattoo placement. This typically came in the form of an incomplete endoscopy report or referral letter [10]. As we have seen from recently published data, the implementation of synoptic reporting substantially increases fidelity to key information, despite the presence of user hesitancy regarding functionality or time requirements [14]. The impetus for implementing a synoptic report for documenting endoscopic localization of colorectal neoplasms was to achieve a similar improvement in report fidelity, and therefore eliminate a major cause of repeat preoperative endoscopy. Participating endoscopists clearly felt that synoptic reporting would improve communication between index endoscopists and operating surgeons, reporting high rates of perceived credibility added to endoscopy reporting and the ability of synoptic reporting to provide necessary surgical planning information. Our future research will measure the effect of our intervention on endoscopy report quality and repeat endoscopy rates.

One concerning outcome of this study is that only half of the participants made regular or occasional use of the new synoptic templates. However, on qualitative analysis, it appears as though the root of this disengagement appears to lay more with an ingrained bias against synoptic templates as a whole, rather than against this particular iteration. This apparent bias is further reflected by participants' low scores for the perceived effect of synoptic report implementation on repeat preoperative endoscopy (3.76/5) and benefits to patient care (3.15/5). Moreover, the majority of study participants made no use of electronic reporting templates before the study and that trend continued beyond the study as well. In prior synoptic report implementation research, participant concerns often arose regarding possibly lack of note detail or inaccurately recorded information, which may have been concerns our participants also held but did not express in their narrative comments [14]. Despite some participants' apparent bias against synoptic report use, there is strong support in the literature for their use to enhance documentation quality [18]. The Achilles heel of the strategic rollout, despite the email and poster distributions, was our failure to successfully advertise the availability of this template for use. The high reported scores of functionality and perceived impact of these updates, contrasted with the relatively low uptake levels, suggest that had the education and distribution been more effective, uptake may have been higher. Those making use of the template found it to be functional and impactful, but more work is required to raise broad awareness and acceptance among the endoscopy community locally.

As strategic plans are made to implement these updated recommendations in a practical and functional way, attention must be paid to the need for continued integration of electronic reporting software to include readily accessible evidence-based synoptic templates integrated with free text options. Once these updates are achieved, increased levels of advertising regarding these updates are required, whether that be electronic (i.e., email), paper-based (infographics and posters), or in-person (multidisciplinary endoscopy rounds). Finally, in addition to making concerted efforts to raise awareness, user training regarding the increased functionality, and potential impact of synoptic templates on clinical practice, outcomes should be recognized as essential to engaging potential new users, improving communication, and reducing rates of preoperative repeat colonoscopy. 

Limitations

The results of this study need to be interpreted in light of certain limitations. Selection and respondent bias are inherent due to the electronic interview study design. We attempted to limit this bias by inviting all endoscopists within the health region to participate, but self-selection to participate does lead to potential bias. The perspectives of our sample population also belong to individuals who work within a single healthcare system and do not necessarily reflect broadly applicable opinions. In particular, the WRHA has a relatively high proportion of surgeon endoscopists compared to other regions in the world, where gastroenterologists may perform a larger portion of endoscopies [10]. Differences in geography and composition allow for a unique analysis but may limit the external applicability of this study. Finally, this study was not powered to detect differences of opinion or practice related to endoscopist specialty.

Future directions

In contrast to respondent enthusiasm regarding the functionality and relevance of these integrated practice updates, a more tempered opinion was observed regarding the ultimate ability such evidence-based synoptic templates will have to decrease the rate of repeat preoperative endoscopy and add true benefit to patient care. As high repeat preoperative endoscopy rates were the initial rationale for this synoptic report's development, future research is needed to determine whether its implementation has had any effect on repeat endoscopy rates. Furthermore, the reasons why some endoscopists may feel less enthusiastic about the prospects of this intervention are important to consider as this research continues. Is there a lack of faith in the fidelity of the endoscopy report that prompts some surgeons to repeat the procedure prior to an operation? Or perhaps some pessimism exists surrounding the true uptake of these recommendations into routine endoscopy practice, despite their perceived benefits. Exploring these and other local cultural practice questions will reveal important information and potential targets for further intervention as we strive to decrease risk, maximize efficient use of healthcare resources, and improve patient care and outcomes related to the management of advanced colorectal neoplasia. 

## Conclusions

The introduction of a synoptic reporting template for evidence-based tattoo localization and documentation of advanced colorectal neoplasms is an important step in the successful implementation of the recently published Delphi-derived, national multidisciplinary consensus recommendations. This study demonstrates that the implementation of a synoptic template, in conjunction with appropriate education, is functional, relevant, and capable of adding credibility and positive influence to endoscopic tattoo localization of advanced colorectal neoplasms. There is a sentiment among some endoscopists that synoptic reporting in general will hinder workflow or add excess time burden, but those interacting with this synoptic reporting tool indicated modest ease of use scores and very low time requirement scores. It also demonstrates the likelihood to improve a vital, yet neglected area of communication between endoscopists and operating surgeons by generating more thorough and accurate colonoscopy reports. However, aggressive advertising and educational campaigns are required to highlight the role that such synoptic reporting templates can play. Endoscopist perspectives indicate that the impact of this standardized synoptic approach in terms of improving patient care and endoscopic resource management by reducing potentially unnecessary repeat preoperative colonoscopies will be important if uptake can be improved and functionality streamlined. Whether improved endoscopy reporting leads to reduced repeat endoscopy is unknown and is an active area of ongoing research.
